# Boosting nutrient starvation-dominated cancer therapy through curcumin-augmented mitochondrial Ca^2+^ overload and obatoclax-mediated autophagy inhibition as supported by a novel nano-modulator GO-Alg@CaP/CO

**DOI:** 10.1186/s12951-022-01439-0

**Published:** 2022-05-12

**Authors:** Xuan Wang, Yunhao Li, Fan Jia, Xinyue Cui, Zian Pan, Yan Wu

**Affiliations:** 1 CAS Key Laboratory for Biomedical Effects of Nanomaterials and Nanosafety, CAS Center for Excellence in Nanoscience, National Center for Nanoscience and Technology, No. 11 First North Road, Zhongguancun, Beijing, 100190 China; 2 Department of General Surgery, Peking Union Medical College Hospital, Peking Union Medical College, Chinese Academy of Medical Sciences, Beijing, 100730 People’s Republic of China; 3 University of Chinese Academy of Sciences, Beijing, 100049 People’s Republic of China

**Keywords:** Cancer metabolism, Curcumin, Mitochondrial Ca^2+^ overload, Autophagy, Starvation therapy, Complementary modality

## Abstract

**Background:**

By hindering energy supply pathway for cancer cells, an alternative therapeutic strategy modality is put forward: tumor starvation therapy. And yet only in this blockade of glucose supply which is far from enough to result in sheer apoptosis of cancer cells.

**Results:**

In an effort to boost nutrient starvation-dominated cancer therapy, here a novel mitochondrial Ca^2+^ modulator Alg@CaP were tailor-made for the immobilization of Glucose oxidase for depriving the intra-tumoral glucose, followed by the loading of Curcumin to augment mitochondrial Ca^2+^ overload to maximize the therapeutic efficiency of cancer starvation therapy via mitochondrial dysfunctions. Also, autophagy inhibitors Obatoclax were synchronously incorporated in this nano-modulator to highlight autophagy inhibition.

**Conclusion:**

Here, a promising complementary modality for the trebling additive efficacy of starvation therapy was described for cutting off the existing energy sources in starvation therapy through Curcumin-augmented mitochondrial Ca^2+^ overload and Obatoclax-mediated autophagy inhibition.

**Graphical Abstract:**

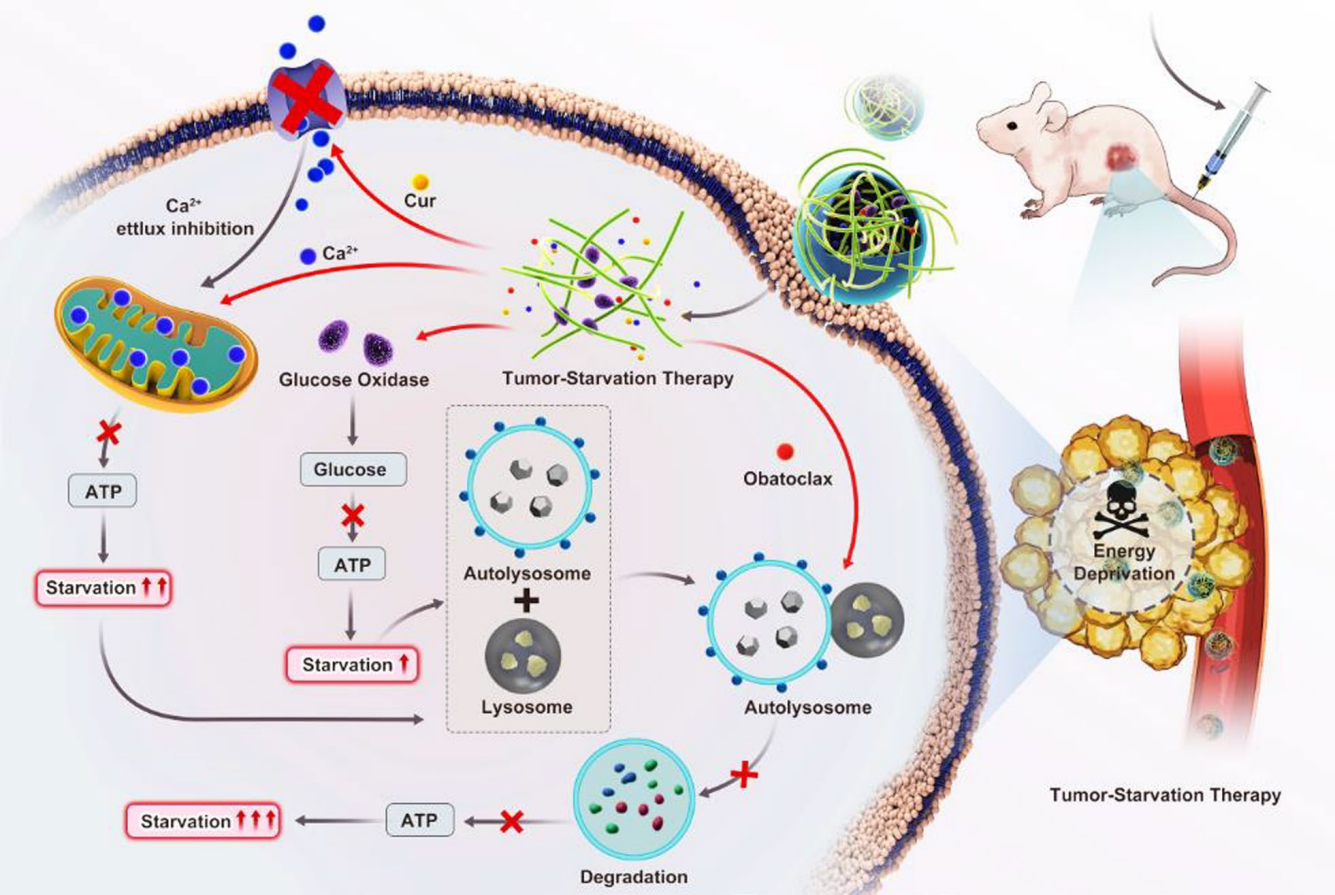

**Supplementary Information:**

The online version contains supplementary material available at 10.1186/s12951-022-01439-0.

## Background

Starvation therapy exerted a promising paradigm for cancer cases through altering the metabolism pathways of cancer cells [1–4]. It is because Warburg pointed out the obvious fact that cancer cells need much more glucose to produce the sufficient energy satisfying the requirements of tumor survival and growth [5, 6]. In this view, glucose oxidase (GO) [7] could be widely used by depriving the intra-tumoral glucose for starvation therapy [8]. And yet only in this blockade of glucose supply which is far from enough to result in sheer apoptosis of cancer cells [9, 10]. Embedded in this fact is the notion that a complementary modality for remarkable additive antitumor effects which have drawn increasing interests in the design of cancer starvation therapy.

Before leaping to construct a combination therapy based on cancer starvation therapy, it might be as well to look more closely to the existing protective mechanism of cancer cells which would compensate for the energy deficiency in the harsh conditions, including starving, organelle damage and other extreme situations [11]. To a certain point, autophagy is defined as an alternate cancer metabolism. Galluzzi et al. [1] pointed out that autophagy could retrieve fuel for ATP synthesis as well as build blocks for essential anabolic reactions in the condition of dwindling nutrient supplies. Thereafter, combination therapy of cancer starvation with autophagy inhibition would be an emerging method for amplifying the therapeutic effects. Yang et al. [11] echoed this notion by suggestive of a synergetic therapy which incorporated an antiglycolytic agent in conjunction with the autophagy inhibitor black phosphorus nanosheet for augmenting tumor-starvation therapy. Such views also have demonstrated in our previous study [12–14].

Moreover, mitochondrion is a key power house of most cells, so it is with cancer cells as well [15]. There have been a vast body of studies involving mitochondria that would commence compensating for the insufficient energy of in harsh conditions of cancer cells through the tricarboxylic acid cycle (TCA) and oxidative phosphorylation [16–19]. Questions have been raised about the impact of mitochondria which might remedy the energy deprivation of starvation therapy [20]. Also, mitochondria are essential for the regulation of the intrinsic pathway of apoptosis [19]. Implicit in this view is an assumption that a novel coordinated method might be sought to maximize the therapeutic efficiency of cancer starvation therapy via mitochondrial dysfunctions.

To date, various methods have been developed and introduced to induce mitochondrial perturbations such as oxidative stress and Ca^2+^ overload [21, 22]. There is increasing attention that mitochondrial Ca^2+^ overload is being advantaged, which is not only because mitochondria are responsible for Ca^2+^ homeostasis [23], but also for the widely used of the Ca^2+^ modulators in anti-tumor field. Questions had been raised about the Ca^2+^ channel which would result in an inferior anticancer effect, since intramitochondrial Ca^2+^ concentration would recover to a normal level through Ca^2+^ excretion [24]. Until recently, Zheng et al. [25] developed a curcumin (Cur)-incorporated CaCO_3_ nanoparticles which could boost Ca^2+^ concentration in mitochondria by Cur, leading to disruption of mitochondrial homeostasis. In reviewing the literature [26–31], the Ca^2+^ modulators such as calcium phosphate (CaP) nanoparticles have also been widely used as multifunctional nano-platforms for Ca^2+^ overload-mediated cancer therapy. Similarly, the correlation between Cur and Ca^2+^ overload was confirmed by Xu et al. Moreover, in accordance with the present results, prior studies have demonstrated that unmodified CaP tended to aggregate to form large particles which was markedly hampered by its instability and poor solubility. The naturally occurring alginate polymers have a wide potential in drug formulation due to their extensive application as food additives and their recognized lack of toxicity. Alginates (Alg), the naturally polysaccharide [32, 33], can be tailor-made to partially alleviate the aggregation of CaP via the affinity between the presence of Ca^2+^ and the carboxylate of the Alginate [34–36]. Moreover, GO could be successfully immobilized with Alginate by taking advantages of the rich functional amino groups.

**Scheme 1:**
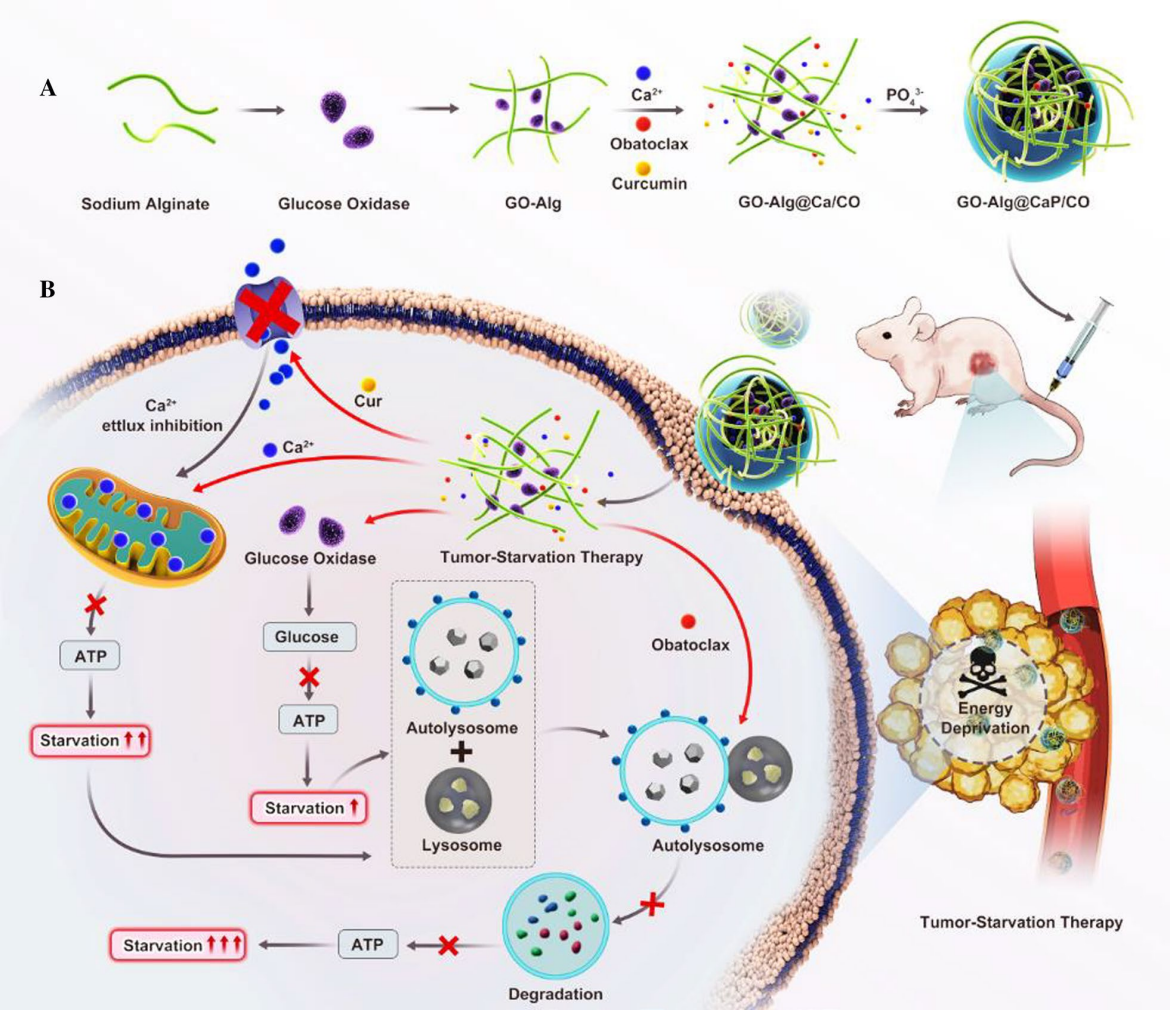
**A** Schematic diagram of the novel nano-modulator GO-Alg@CaP/CO. **B** Schematic illustration of the promising complementary modality for the trebling additive efficacy of starvation therapy which was described for cutting off the existing energy sources in starvation therapy through curcumin-augmented mitochondrial Ca^2+^ overload and obatoclax-mediated autophagy inhibition

Based on these views, to carry out the study of boosting nutrient starvation-dominated cancer therapy has become essential. The aim of this study is to establish a novel nano-modulator based on the GO-Alg@CaP/CO as the following steps in scheme 1A. (1) GO would be immobilized with Alg according to the facile reaction between –NH_2_ and –COOH. (2) GO-Alg was involved with CaP to suppress the aggregation of CaP via the affinity between the presence of Ca^2+^ and Alg. (3) Cur and autophagy inhibitors Obatoclax were synchronously incorporated in this nano-modulator by the coprecipitated method to highlight the mitochondrial Ca^2+^ overload and autophagy inhibition. Taken together, GO-Alg@CaP/CO could cut off the existing energy sources in starvation therapy through curcumin-augmented mitochondrial Ca^2+^ overload and autophagy inhibition, which would be a promising complementary modality for the trebling additive efficacy of starvation therapy (Scheme 1B).

## Results and discussion

### Characterizations of synthesized GO-Alg@CaP/CO

**Fig. 1:**
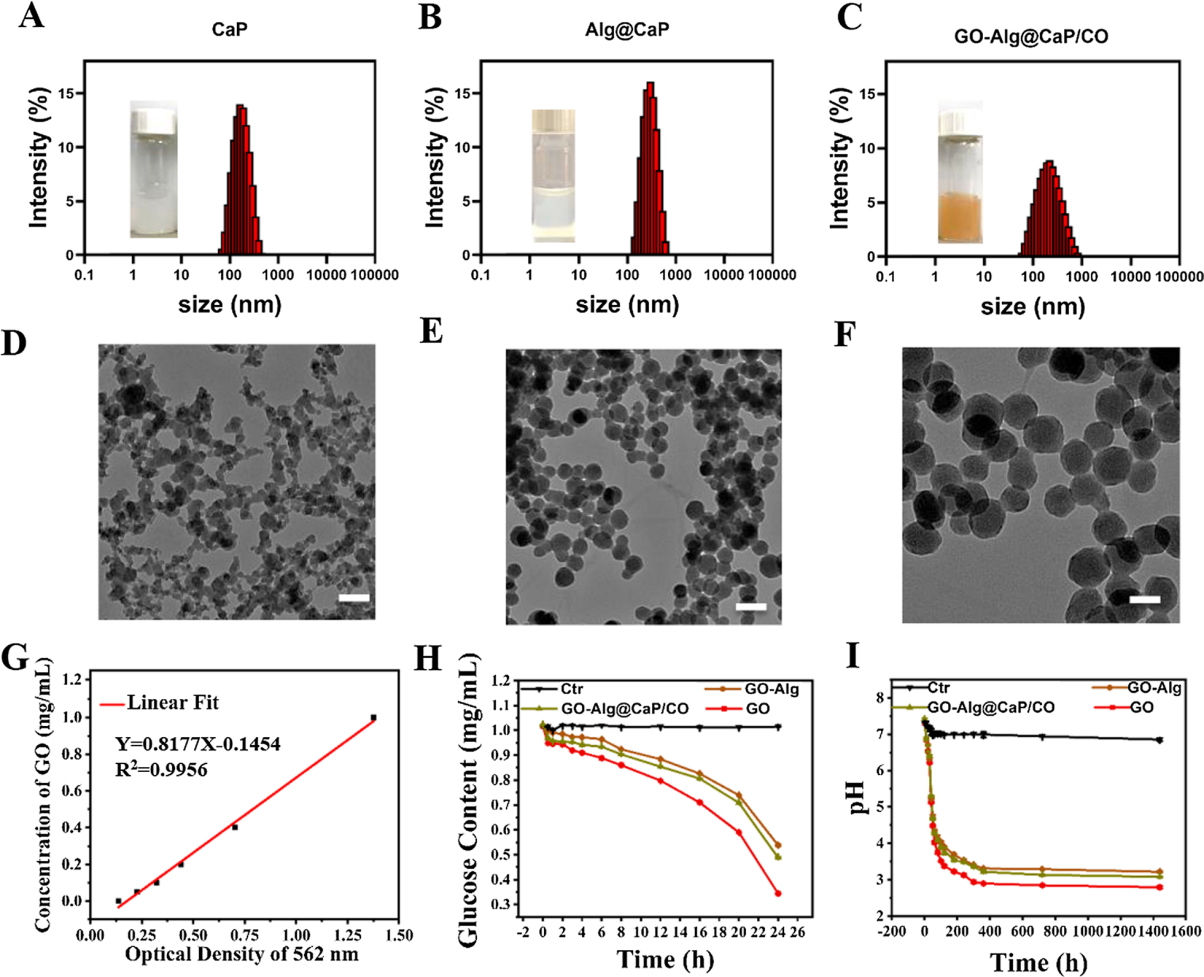
Particle sizes of **A** CaP, **B** Alg@CaP and **C** GO-Alg@CaP/CO. These insets showed the images of the corresponding aqueous solution. Transmission electron microscopy (TEM) of **D** CaP, **E** Alg@CaP and **F** GO-Alg@CaP/CO (scale bars were 200 nm). **G** The standard content curves of GO based on BCA assays. **H** Variation of glucose concentration and **I** pH value in glucose solution (1 mg/mL) after the addition of GO, GO-Alg and GO-Alg@CaP/CO with the identical GO concentration (1 mg/mL)

At the acidic conditions, GO was capable of immobilizing on the abundant carboxyl groups of Alg via a facile EDC/NHS-induced covalent reaction. First, BCA assays was established to access the successfully immobilization of GO. Figure 1G provided the standard linear calibration curve and GO concentration can be calculated from the following formula (Eq. 1) (R^2^ = 0.9956):

1 $${\text{C}} \left( {{\text{GO}}} \right)\left( {\frac{{{\text{mg}}}}{{{\text{mL}}}}} \right) = 0.8177 \times {\text{OD}} - 0.1454.$$

After diluted 10 times, the OD (Optical Density) of GO-Alg was 0.28 and GO concentration was then identified with the above formula as 0.084 mg/mL. Eventually, the immobilization efficiency (IE) of GO was calculated as 84% with the following formula (Eq. 2):

2 $${\text{IE}}\left( {{\text{GO}}} \right)\left( \% \right) = \frac{{{\text{GO}}\;{\text{quantified}}\;{\text{in}}\;{\text{the}}\;{\text{GO-Alg}}\;{\text{solution}} \left( {{\text{mg}}} \right)}}{{{\text{Total}}\;{\text{GO}}\;{\text{added}} \left( {{\text{mg}}} \right)}}.$$

The results of the correlational analysis were echoed that GO could be easily and effectively immobilized on Alg. To carry this idea one step further, amide N–H stretch at 3700–3500 cm^−1^, amide C=O stretch at 1690–1630 cm^−1^, and amide I band and II band at 1500–1560 cm^−1^ were shown in GO-Alg at FT-IR spectrum (Additional file 1: Fig. S1).

The next section of the results was concerned with GO-Alg@CaP. It can be seen from Additional file 1: Table S1, there were clear trends of increasing in the average particle size and decreasing in the zeta potential of GO-Alg@CaP with different ratios (GO-Alg/CaP) ranged from 0/10 to 50/10. Overall, an implication of this was the possibility that GO-Alg could be successfully involved with CaP due to the affinity between Ca^2+^ and Alg. This view was further confirmed in the GO-Alg@CaP/CO spectra (Additional file 1: Fig. S1), the bands ranged from 1400–1700 cm^−1^ and 3000–3500 cm^−1^ were attributed to GO-Alg. The bands appearing in the range of 800–1200 cm^−1^ could be ascribed to –PO_4_^3−^ bonds of CaP. Similarly, the characteristic peaks of Alg were also shown in the Alg-CaP spectra (Additional file 1: Fig. S1). Additional file 1: Table S1 also presented a random polydispersity index (PDI) which should be considered. The sizes, zeta potentials and PDI were all profoundly large when the ratio was over 5/10 due to the high viscosity of the Alg, which was not neglected for Alg to produce larger emulsion droplets leading to larger particles. Eventually, 5/10 (GO-Alg/CaP) was chosen for he further experiments with an ideal property. Similarly, the final GO concentration of GO-Alg@CaP was also confirmed with BCA assay as 80% IE.

Prior studies had noted the high drug loading efficiency of CaP. GO-Alg@CaP also endowed a large capacity for the drug loading which was not restricted in the GO-Alg coating. Moreover, the percentages of Cur and Obatoclax entrapped into GO-Alg@CaP were both almost 90%. A probable explanation was that GO-Alg interaction with CaP provided an excellent natural polysaccharide coating that also elicited a special adhesive affinity to drugs. And Additional file 1: Table S2 had set out the basic characterizations of the final nano-formulations, including that GO-Alg@CaP/CO had a size distribution of 204.1 nm with a PDI of 0.227. Even though larger particle size of GO-Alg@CaP could be also obtained from Fig. 1A–F in accordance with previous results. On the contrary, for the morphology of GO-Alg@CaP/CO, it was almost certain from Fig. 1D, E that GO-Alg could partially control the irregular aggregation of CaP, suggestive of the regular spherical shape. We determined the stability of GO-Alg@CaP/CO under biological conditions during 5 days as following. As exhibited in Additional file 1: Fig. S3, the size change of GO-Alg@Cap/CO was almost negligible at physiological conditions of pH 7.4 for 5 days, suggestive of good stability for the intravenous injection. Moreover, Additional file 1: Fig S4A, B showed that only 35% of Obatoclax and 28% Curcumin were released from GO-Alg@CaP/CO at the pH 7.4 over 240 h, while nearly 50% of Obatoclax and Curcumin could be released at pH 5.2, indicating that the pH-responsive dissociation of CaP which was in accordance with our previous studies. Taken together, these results held that GO-Alg@CaP/CO would be promising nanocomplexes for the following application.

### Catalytic ability measurement of synthesized GO-Alg@CaP/CO

The glucose content was established by a DNS method through a linear fit curve between glucose concentration and UV–Vis absorption (540 nm) (Additional file 1: Fig. S2). By incubating glucose solution with different preparations containing the identical GO concentration, as shown in Fig. 1H, I, the glucose content and pH value demonstrated a clear trend of decreasing with time extended, revealing the successful conversion of glucose into gluconic acid together with pH value dropped from ~ 7.4 to ~ 3.0 and glucose decrease from 1 to ~ 0.4 mg/mL, respectively. Another important finding was that GO-Alg and GO-Alg@CaP/CO exhibited similar catalytic efficacy but suggested weaker activity in comparison with GO, indicating that GO interaction with Alg would alter the catalytic activity of GO. Therefore, incomplete reaction might be a major factor because the immobilized GO could not completely be involved with glucose. In this view, these changes were reasonable without significant difference and the activities of GO in GO-Alg and GO-Alg@CaP/CO were also well preserved with quickly decreased pH ranged from 7.4 to 3.2 and gradually dropped glucose from 1 to 0.48 mg/mL.

### The cellular uptake of GO-Alg@CaP/C

As elicited in Additional file 1: Figs. S5, S6, the cells treated with Cur still showed weaker green fluorescence compared with GO-Alg@CaP/C for different time points. Moreover, the co-localized green (GO-Alg@CaP/C) with red (lysosomes) fluorescent after 6 h noted that the cellular uptake of GO-Alg@CaP/C was attributed to endocytosis. It seemed possible that nano-complexes could improve the solubility of Cur which might facilitated the final cellular uptake, indicating that nano-complexes GO-Alg@CaP/C endowed increased cellular internalization efficiency.

### In vitro Ca^2+^ production by GO-Alg@CaP/CO

**Fig. 2:**
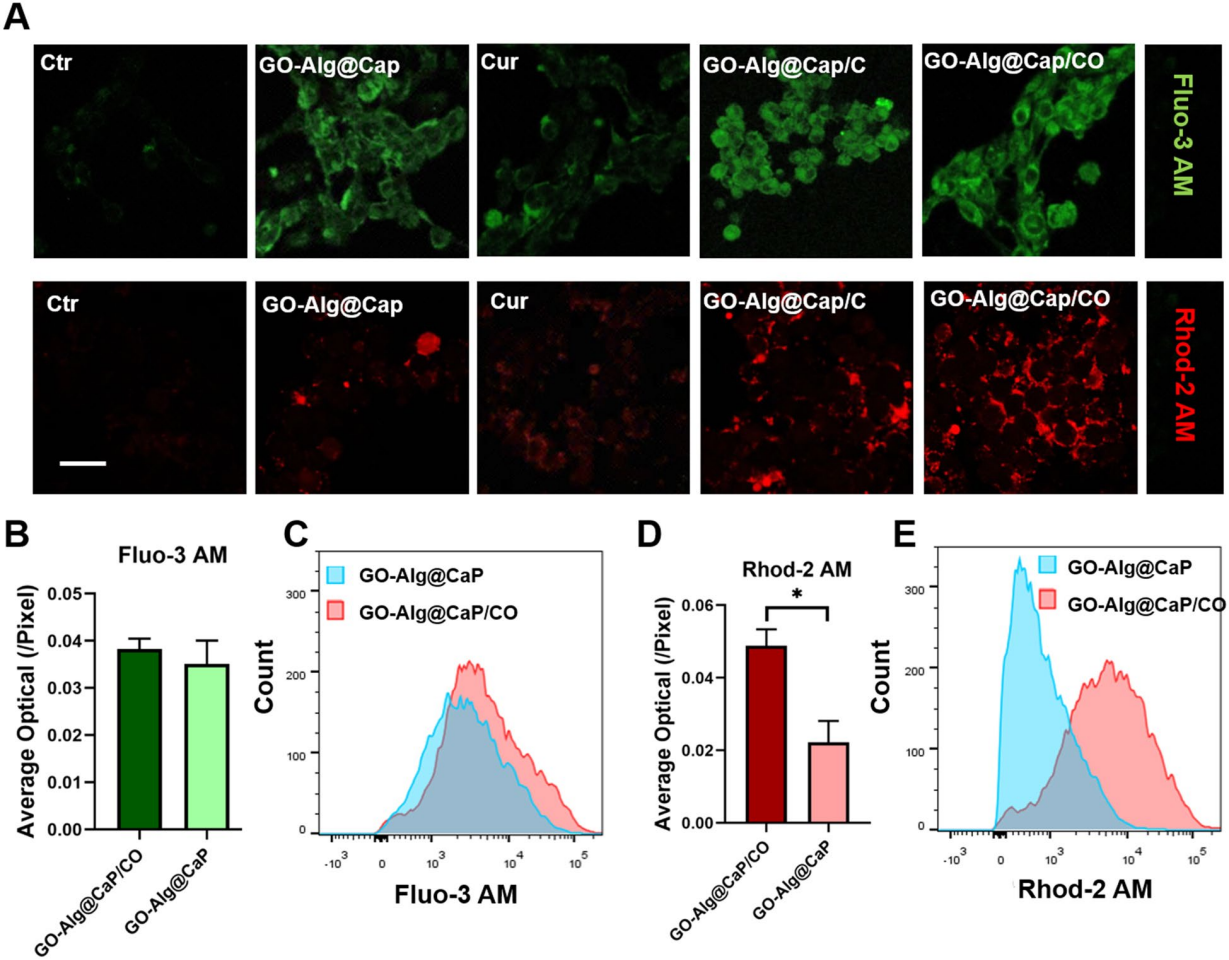
Disruption of intramitochondrial Ca^2+^ homeostasis **A** intracellular Ca^2+^ production and mitochondrial Ca^2+^ concentrations of 4T1 cells treated with different preparations for 6 h. Scale bars were 50 μm. **B**–**E** Flow cytometry results of 4T1 cells incubated with GO-Alg@CaP and GO-Alg@CaP/C. Data are mean ± SD, n = 3; *P < 0.05, **P < 0.01 and ***P < 0.001

**Fig. 3:**
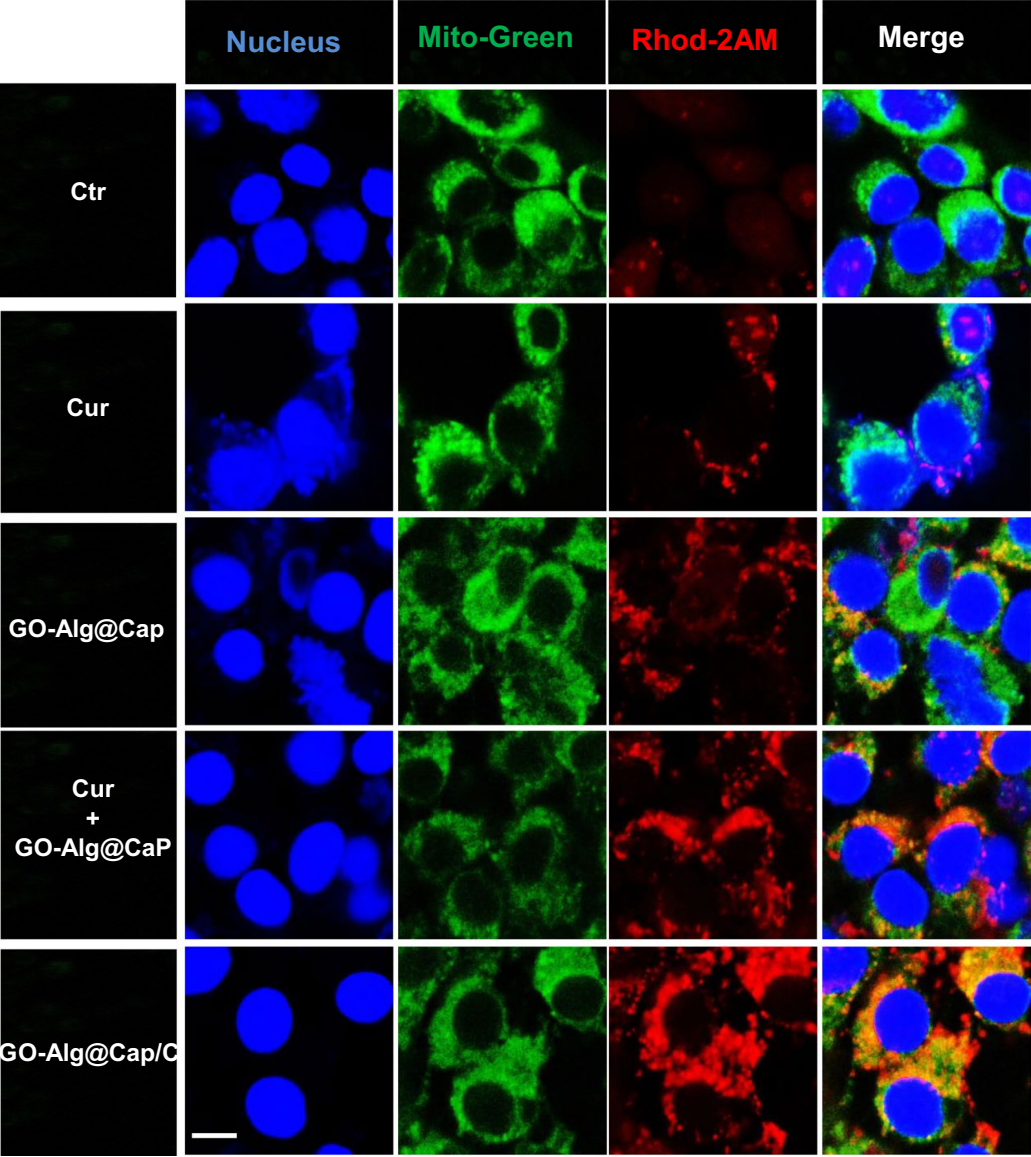
Biodistribution of Ca^2+^ in mitochondria in 4T1 cells after incubation with different preparations for 6 h. The nucleus and mitochondria were stained with Hoechst 33342 and Mito-Green, respectively. The scale bar was 10 μm

Prior to detecting Ca^2+^ in mitochondria, free Ca^2+^ in 4T1 cells was stained with Fluo-3 AM to be visualized by CLSM. It was because there had been an apparent increase of green fluorescence intensity in all nano-complexes with CaP. Figure 2 revealed that CaP could give rise to high free Ca^2+^ concentration in 4T1 cells compared to free drugs and ctr groups. As expected, CaP could be collapsed in cells and eventually lead to producing intracellular overmuch free ionic calcium. Based on the above analysis, the intramitochondrial Ca^2+^ concentration alteration was then visualized by CLSM with the help of a Rhod-2 AM staining assay. In particular, it was somewhat surprising that the observed difference between GO-Alg@CaP/CO group (without Cur) and GO-Alg@CaP group (with Cur) was significant. Although GO-Alg@CaP/CO group (without Cur) and GO-Alg@CaP group (with Cur) endowed with similar intracellular ionic calcium in the Fig. 2B, C, there was a significant difference in above two groups by flow cytometry in Fig. 2D, E, indicating that Cur might attribute to inhibit expelling of Ca^2+^ and induce the mitochondrial Ca^2+^ accumulation. It was also verified in the relevant studies that Cur might attribute to inhibit expelling of Ca^2+^ and induce the mitochondrial Ca^2+^ accumulation^24^. To further illustrate these findings, the co-localization of Ca^2+^ and mitochondria was clearly demonstrated in Fig. 3. Notwithstanding the high red fluorescence intensity, no significant co-localization of Ca^2+^ and mitochondria in GO-Alg@CaP group was observed which was consistent with above findings. Furthermore, there were distinctly overlaps between green fluorescent (mitochondria) and red fluorescent (Ca^2+^) in both GO-Alg@CaP/C and GO-Alg@CaP+Cur, highlighting a noticeable increase of free Ca^2+^ in mitochondria induced by Cur.

### Mitochondrial disfunction induced by GO-Alg@CaP/CO

To carry these results one step further, JC-1 staining was used to visualize mitochondrial membrane potentials (MMP). High MMP could be associated with J-aggregates shown as red fluorescence. On the contrary, low MMP would represent a monomer with green fluorescence. To assess MMP, the relative levels of green and red fluorescence intensities were compared in different preparations. As depicted in Additional file 1: Fig. S7, different nano-complexes exhibited lower red fluorescence than free drugs and ctr groups. Moreover, GO-Alg@CaP/CO group pervaded the remarkable increased green fluorescence suggesting the lowest MMP, which was similar with the analysis of free Ca^2+^ in mitochondria.

### Autophagy inhibition evaluation of GO-Alg@CaP/CO

**Fig. 4:**
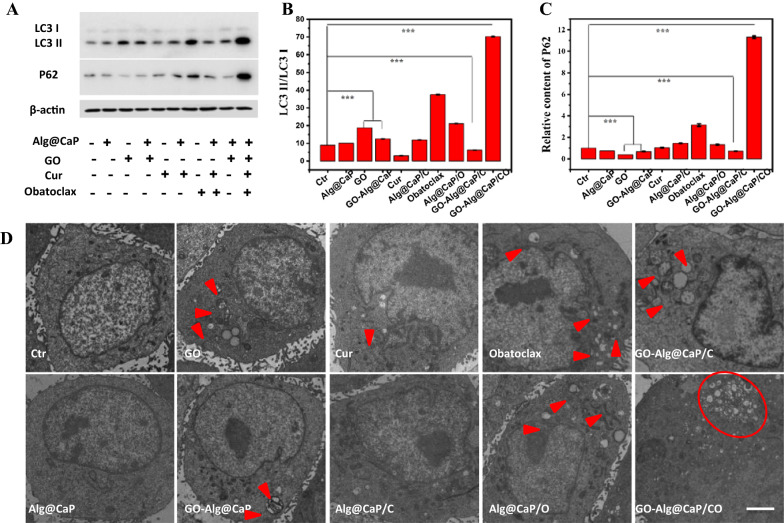
**A** P62, LC3-I, and LC3-II expression in 4T1 cells with a western-blotting analysis under different groups. Matching gray-scale analysis of **B** LC3-II/LC3-I and **C** P62. **D** TEM images of autophagosome. Red arrows and circles implied the autophagosome. Data are mean ± SD, n = 3; *P < 0.05, **P < 0.01, and ***P < 0.001 vs control. The scale bar was 5 μm

The complex correlation of autophagy and energy deficiency encouraged us to further investigate autophagic responses after different treatments. As a typical autophagosomal biomarker, both LC3 and P62 were widely considered to reveal the autophagy level in cells. The relationship of LC3-II and autophagy was complex and intertwined, it can be exclusively determined that the autophagy level was inversely related to P62 on the basis of the improved level of LC3-II. The observed increase of LC3 II/LC3 I and reduction of P62 in GO and GO-Alg@CaP groups were visualized in Fig. 4 and the repeated experiments were shown in Additional file 2. It can therefore be assumed that the high level of autophagy was stimulated in starvation therapy. Moreover, Alg@CaP and Alg@CaP/C groups also pointed out slight accumulation of autophagosomes together with the upregulation of LC3 II. It was possible to hypothesize that harsh conditions including mitochondrial Ca^2+^ overload would improve autophagy level as well. More importantly, very little autophagosomes were found in these groups (Fig. 4D), which was in agreement with above discussion. Comparatively, significant increase of LC3 II/LC3 I and P62 was also observed in Obatoclax treated groups (Fig. 4A–C), characteristic of the lower autophagy level. As shown in Fig. 4D, the increase in the quantity of autophagosomes were both observed in GO-Alg@CaP group and GO-Alg@CaP/CO group and the latter exhibited the most autophagosomes absolutely. Taken together, above results further revealed that GO-Alg@CaP enhanced the accumulation of autophagosomes by the activation of autophagic flux instead of blocking it. On the contrary, GO-Alg@CaP/CO could cause quantity of autophagosomes retention by blocking lysosomal degradation. These results were consistent with our previous studies and suggested that GO-Alg@CaP/CO could suppress the existing protective mechanism such as autophagy which would compensate for the energy deficiency in the harsh conditions.

### In vitro cytotoxicity and apoptosis of GO-Alg@CaP/CO

**Fig. 5:**
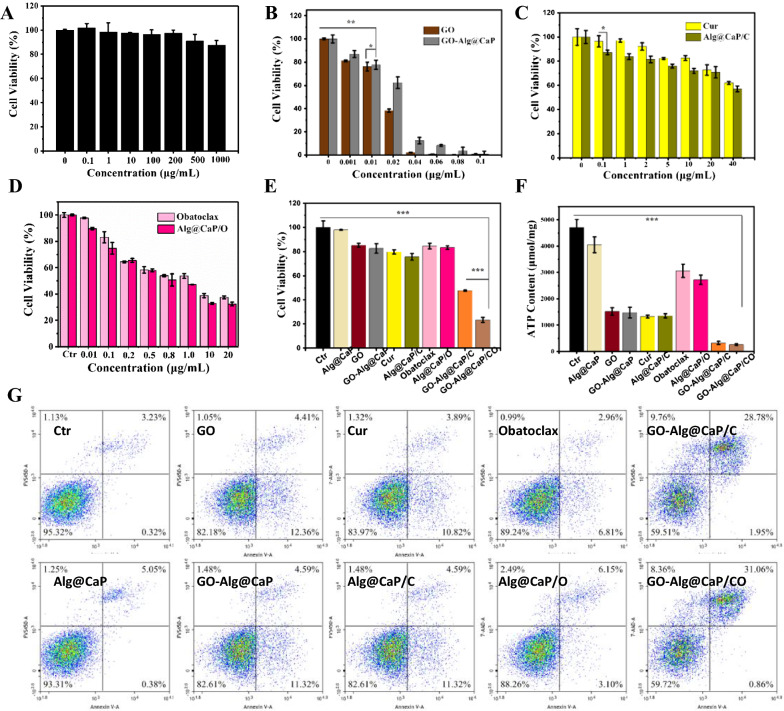
**A** Relative viability of 4T1 cancer cells cultured with blank carriers (Alg@CaP) at different concentrations. Relative viability of 4T1 cancer cells cultured with different treatments. **B** GO and GO-Alg@CaP, **C** Cur and Alg@CaP/C, and **D** obatoclax and Alg@CaP/O. **E** Relative viability of 4T1 cancer cells cultured with different preparations at desired concentrations for 24 h. **F** Intracellular ATP content analysis of 4T1 cancer cells cultured with different preparations at desired concentrations for 48 h. **G** Apoptosis assays of 4T1 cells in different groups with a flow cytometric analysis. Data are mean ± SD, n = 3; *P < 0.05, **P < 0.01 and ***P < 0.001 vs. control

Figure 5A first pointed out that blank carriers (Alg@CaP) were almost non-toxic with around 90–100% cell viability. Thereafter, the concentration gradient of each and every drug was set up to compare the efficacy differentiation between free drugs and nano-complexes. In general, the negative correlation between the cell viability and the drug concentration were apparently presented in Fig. 5B–D. A comparison of the free drugs and nano-complexes revealed striking discussions as following. Cell viability of GO-Alg@CaP reported significantly more than GO at GO concentration of 0.02 μg/mL (Fig. 5B) which was consistent with previous catalytic ability analysis. It was because the GO-Alg@CaP endowed lower catalytic ability to result in much less cell death than GO. From the graph in Fig. 5B, both GO and GO-Alg@CaP would elicit a sharp lethality more than 85% at the desired concentration of GO (0.04 μg/mL), revealing that the glucose of cells was almost depleted by GO inducing cytotoxicity. In order to comprehensively assess the boosting nutrient starvation efficacy, lower GO concentration (0.01 μg/mL, 20% inhibitory concentration) was considered to eliminate the sole contribution to cytotoxicity by GO. Compared with Cur and Alg@CaP/C, the well efficacy of Alg@CaP/C was demonstrated (Fig. 5C) which was also in accordance with above better cellular uptake. Similarly, 5 μg/mL of Cur (20% inhibitory concentration) was selected for the following experiments. As for Obatoclax and Alg@CaP/O, negligible cytotoxicity with 0.01 μg/mL was found (Fig. 5C) which could be identified for further evaluation. For further emphasizing the augmented efficiency of GO-Alg@CaP/CO, the evaluation of cytotoxicity was finally established for different treatments. The observed highest lethality in GO-Alg@CaP/CO treated cells with 18% cell viability which could be attributed to the combined effects of several treatments (Fig. 5E). The differences between the sum of each treatment and GO-Alg@CaP/CO also suggested this view.

The results obtained from Fig. 5G pointed out that each single treated cells’ late apoptosis was lower than ~ 5%. However, we found that about 31.06% of GO-Alg@CaP/CO treated cells died of late apoptosis which was highest upon all groups, indicating that this high efficacy was stemmed from complex synergistic effects upon substances such as GO, Cur, CaP and Obatoclax. In accordance with above cytotoxicity evaluation, the apoptosis of GO-Alg@CaP/CO and GO-Alg@CaP/C were also not only the summarization of each single treatment. Together these results provided important insights into the GO-Alg@CaP/CO for enhanced efficacy.

To clarify energy metabolism perturbations, the results obtained from the preliminary quantified intracellular ATP levels were summarized in Fig. 5F. Distinct decreases of ATP production were observed in GO, GO-Alg@CaP, while no significant change was observed in Alg@CaP group, demonstrating that blockade of glucose supply by GO was capable of exacerbating of energy deprivation. Also, Cur and Alg@CaP/C groups could lead to mitochondrial dysfunction through Ca^2+^ efflux inhibition via Cur which would cut off the energy sources from mitochondria consistent with the previous discussion, revealing a considerable decrease in the ATP level. The graph presented a slight fall in the ATP level at Obatoclax and Alg@CaP/O groups, which was stemmed from another energy deprivation source (autophagy inhibition) caused by Obatoclax. As a consequence of the trebling additive efficacy of energy depletes, it appeared that a sharp drop in the ATP level of GO-Alg@CP/CO group.

### In vivo antitumor efficacy of GO-Alg@CaP/CO

The hemocompatibility was first investigated to confirm in vivo application of GO-Alg@CaP/CO. As shown in Additional file 1: Fig. S8, even high concentration of nano-complexes around 1000 μg/mL was endowed with a relative low hemolysis rate of 0.87% after 24 h incubated, indicating extraordinary hemocompatibility.

**Fig. 6:**
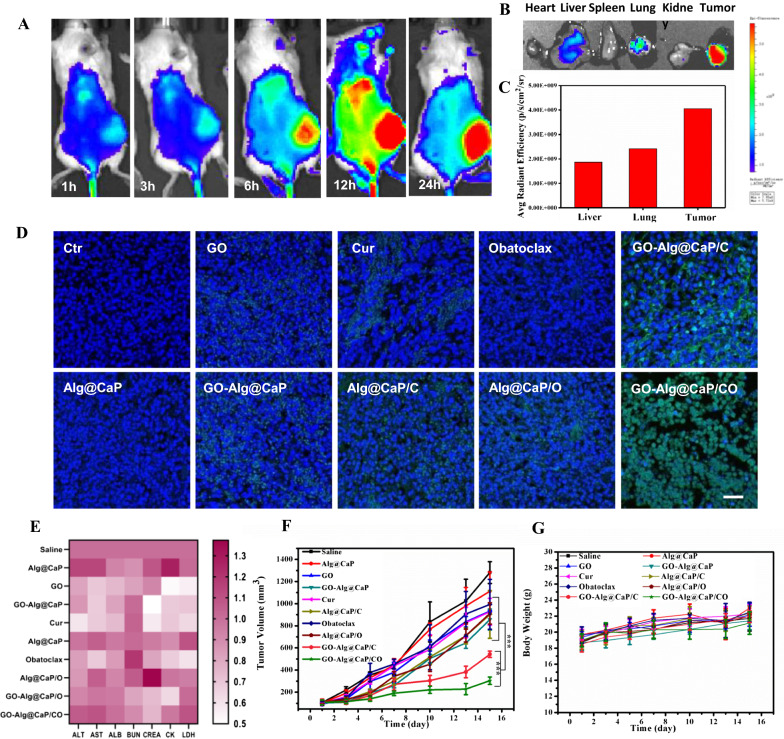
**A** In vivo fluorescence images of 4T1 tumor-bearing mice that were intravenously injected with Alg@CaP/I at different time points and **B** ex vivo fluorescence images of major organs and tumors at 24 h post injection. **C** Histogram analysis of relative average radiant efficiency of liver, lungs, and tumor. **D** TUNEL-stained tumor slices collected from 4T1 tumor-bearing mice after treatments for 15 days. The scale bar was 50 μm. **E** Blood analysis of mice at 15 days after various treatments. **F** Relative volume changes and **G** mice body weight changes in different groups. Data are mean ± SD, n = 5; *P < 0.05, **P < 0.01, and ***P < 0.001 vs. control

Substantial cellular uptake results about GO-Alg@CaP/CO led us to further explore in vivo distribution of nano-complexes. IR780 was loaded as a fluorescent marker in nano-complexes and then injected into tumor-bearing mice via tail vein. The repeated experiments were shown in Additional file 1: Fig. S17, and it was apparent that the accumulation of nano-complexes at tumor sites was observed. There was a clear trend of increasing of fluorescence intensity at tumor sites within 12 h but a slight decreasing after 24 h. In response to these results, the fluorescence intensities of organs and tumor were detected ex vivo (Fig. 6B, C). A clear high retention of GO-Alg@CaP/CO at tumor sites was noted, revealing their efficient passive EPR effect (enhanced permeability and retention effect) at tumor sites. Overall, this was an important criterion for the next therapeutic evaluation of GO-Alg@CaP/CO.

Prior cellular studies had shown synergistic therapeutic effects as a consequence of the trebling additive efficacy of energy depletes of GO-Alg@CaP/CO. To further evaluate their therapeutic effect in vivo, 4T1 tumor-bearing mice were used. Fast increase of the tumor volumes in saline and single treatment groups was visualized in Fig. 6F, Additional file 1: Figs. S9 and S11, while the growth of tumors in GO-Alg@CaP/C and GO-Alg@CaP/CO groups was slightly restrained as considerable energy depletes routes boosting starvation therapy. We tried to detect the ATP in tumor issues after GO-Alg@CaP/CO treatment in Additional file 1: Fig. S12. In accordance with precious in vitro ATP assay, distinct decreases of ATP production were observed after 15 days GO-Alg@CaP/CO treatment, demonstrating that GO-Alg@CaP/CO was capable of exacerbating of energy deprivation in tumor issues. Solid evidence about efficient synergistic starvation therapy (tumor inhibition rate of 85% for GO-Alg@CaP/CO group) was found from the above graph which was in agreement with the previous cellular results, demonstrating that the blockage of autophagy by Obatoclax could further potentiate energy deprivation in the tumor harsh conditions. We further provided the protein level of LC3 and P62 in tumor after nanocomposites treatments (GO-Alg@CaP and GO-Alg@CaP/CO) to show solid evidence for autophagy evaluation in vivo. As shown in Additional file 1: Fig. S13, slight decrease of P62 and significant increase of LC3 II revealed the high level of autophagy in starvation therapy. Moreover, both significant increase of LC3 II and P62 were also observed in GO-Alg@CaP/CO group, indicating the autophagy was effectively inhibited in accordance with cellular results.

**Fig. 7:**
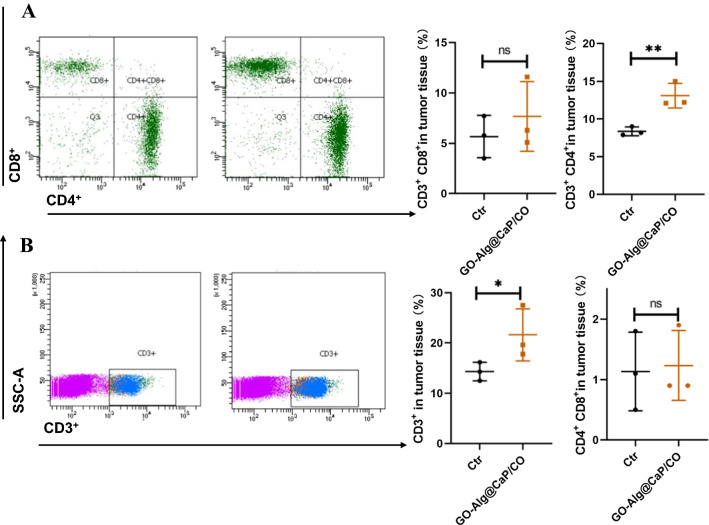
**A** Representative flow cytometric analysis (left) and quantification (right) of CD3^+^CD8^+^ and CD3^+^CD4^+^ T cells in the tumor. **B** Representative flow cytometric analysis gating on CD3^+^ cells (left) and relative quantification (right) of CD4^+^CD8^+^ T cells and CD3^+^cells. Data are mean ± SD (n = 3). *P < 0.05; **P < 0.01; ***P < 0.001

TUNEL immunofluorescence staining and H&E staining were applied in the follow-up experiment of the therapeutic evaluation. As illustrated in Fig. 6D, GO-Alg@CaP/CO group exhibited highest green fluorescence intensity (revealing dead cells) than other groups, proving the remarkable tumor cell apoptosis. Furthermore, very little cell nucleus was found in GO-Alg@CaP/CO group form the Additional file 1: Fig. S10, demonstrating the same conclusions with the above results. We provided H&E staining images of whole organs (Heart, Liver, Spleen, Lung and Kidney) after treatment in Additional file 1: Fig. S14 and short-term short-term blood biochemical assay data such as 24 h and 7 days after nanoparticle injection in Additional file 1: Fig. S15. The H&E staining of the major organs showed no obvious physiological morphology changes in mice. Delightedly, no evident changes were demonstrated in various serum biochemistry indexes including Liver Function and Renal Function. All evidence suggested that the nanocomposites GO-Alg@CaP/CO would be safely administered for tumor therapy. In the end, no significant fluctuations among mice weight and blood biochemical data were observed in all groups (Fig. 6E, G), confirming negligible toxic and side effects. We added flow analysis in spleen to confirm the biosafety of GO-Alg@CaP/CO. Remarkably, GO-Alg@CaP/CO treatment caused an increase in tumor-infiltrating lymphocytes (TILs, CD3^+^), especially regulatory T cells (Tregs) including CD4^+^, CD8^+^ and CD4^+^CD8^+^ within the tumor (Fig. 7). CD4^+^/CD8^+^. The increased Tregs in the distant tumor could trigger antitumor immunity which would facilitate tumor treatment of GO-Alg@CaP/CO. And we further provide the ratio of CD4^+^/CD8^+^ in Additional file 1: Fig. S16, there was no significant change between Ctr group and GO-Alg@CaP/CO, indicating no obvious damage in immune system after treatment.

## Conclusion

In summary, efforts are dedicated, for the first time, to combining mitochondrial disruption and autophagy inhibition for the trebling additive efficacy of starvation therapy based on a novel nanomodulors GO-Alg@CaP/CO. The local glucose at tumor sites was chiefly consumed by GO to achieve starvation therapy. In response to enhance GO-mediated starvation therapeutic efficiency, a Cur-incorporated CaP could boost Ca^2+^ concentration in mitochondria by Cur, leading to disruption of mitochondrial homeostasis. Meanwhile, Obatoclax was synchronously incorporated in this nanomodulors by the coprecipitated method to highlight autophagy inhibition. Data were gathered from in vitro and in vivo experiments showed great biocompatibility and biosafety of GO-Alg@CaP/CO. The studies presented thus far provide evidence that GO-Alg@CaP/CO could cut off the existing energy sources in starvation therapy through Curcumin-augmented mitochondrial Ca^2+^ overload and Obatoclax-mediated autophagy inhibition, which would be a promising complementary modality for the trebling additive efficacy of starvation therapy.

## Method and materials

### Materials

Sodium alginate (Alg), sodium polyphosphate (TPP) and calcium chloride (CaCl_2_) were got from Beijing Chemical Reagent, China. Glucose oxidase (GO) was obtained from Sigma-Aldrich, USA. Curcumin (Cur) was purchased from Macklin, China. Obatoclax was purchased from Beyotime, China. IR780 was obtained from Alfa Aesar, Ward Hill, USA. Cell counting kit-8 was purchased from Dojindo, Japan. Dulbecco’s modified Eagle’s medium (DMEM) and penicillin/streptomycin were purchased from Gibco, Thermo Fisher Scientific, USA. Fetal bovine serum was obtained from Wisent, Canada. Lysosome-green and Hoechst33342 were obtained from Dojindo, Japan. Fluorescent probes including Mito-green, Fluo-3AM Ca^2+^ and Rhod-2AM were purchased from Beyotime, China.

### Synthesis of GO-Alg

GO-Alg was facile prepared as the following steps according to the previous report [12]. 0.1 mL of EDC (20 mM), 0.1 mL of NHS (20 mM) and 10 mL of Alg (1 mg mL^−1^) were mixed and gently shaken at 37 °C for 15 min. Subsequently, 2 mL of GO (5 mg mL^−1^) were introduced to the above solution for another 2 h. Finally, GO-Alg complexes were dialyzed (MWCO = 3.5 kDa) with ultrapure water for 24 h and obtained by lyophilization for further use. Bicinchoninic acid (BCA) protein assay was used to identify the concentration of GO.

### Synthesis and characterizations of GO-Alg@CaP/CO

To induce GO-Alg associated with Ca^2+^, CaCl_2_ solution was added into a stirring GO-Alg solution and equilibrated for 12 h. Cur and Obatoclax were first dispersed in DMSO and then added into the above solution dropwise under stirring. An aqueous solution of TPP was subsequently dropped into the above mixture slowly and then stirred for another 6 h to stimulate mineralization. GO-Alg@CaP was finally collected by centrifugation and dialyzed for 3 days to completely remove unreacted substances and organic solvents. The drug loading and encapsulate efficiency were detected by HPLC method. BCA protein assay was used to confirm the final concentration of GO. Fourier transformed infrared spectrum (FTIR) was used to confirm the corresponding groups of GO-Alg@CaP/CO. The dynamic light scattering (DLS) measurement was carried out with a Zeta Sizer Nano Nano-ZS (Malvern Instruments, Malvern, UK) to observe the properties of GO-Alg@CaP/CO. Transimission electronic microscopy (TEM, Hitachi, Ht-7700, Japan) were selected to record the morphology of GO-Alg@CaP/CO.

### Catalytic ability measurement

The variations of glucose and pH would be detected to confirm the catalytic activity of GO-Alg@CaP/CO. Briefly, 1 mL of GO-Alg@CaP/CO (GO concentration was set at 1 mg/mL) was added to 20 mL DMEM medium (glucose concentration was identified with 1 mg mL^−1^). Moreover, GO and GO-Alg were also judiciously set to compare the activity with GO-Alg@CaP/CO. Then, a 3,5-dinitrosalicylic acid (DNS) method [7, 37, 38] was used to detect the absorption at 540 nm via the UV–Vis spectroscopy (Persee, TU-1810, China) and a real-time pH meter were used to monitor the glucose content and pH variation over time, respectively.

### Cell culture and tumor cellular uptake

4T1 breast cancer cells were cultured in DMEM medium containing 10% FBS and 1% penicillin/streptomycin at 37 °C under 5% CO_2_ according to the standardized procedure. The green fluorescent of Cur was utilized to monitor the nano-complexes in vitro tracking. 4T1 cells were briefly incubated with Cur and GO-Alg@CP/C for different desired time points such as 0.5 h, 2 h, and 6 h, respectively. Thereafter, cells were washed thrice by using phosphate buffered saline (PBS) and then stained with Lyso-Tracker Red for another 15 min. Eventually, the cells were stained with Hoechst 33342 after thrice washed by PBS. Confocal laser scan microscopy (CLSM, Perkin Elmer, Ultra View Vox system, USA) was conducted to observe the cellular uptake of nanocomplexes.

### In vitro cytotoxicity and apoptosis assay

The previous recommended CCK-8 assay was conducted to evaluate the cytotoxicity of different preparation in 4T1 cells. Briefly, 4T1 cells were seeded in a 96-well plate at a density of 5 × 10^3^ cells per well and cultured in DMEM medium for 24 h. After that, the old medium was removed and added with different treatments. After another 24 h of incubation, CCK-8 reagent was added to each well and incubated for desired time points. The absorbance at 450 nm was subsequently measured by a microplate reader to confirm cell viability.

The apoptosis assay was carried out by a flow cytometry (NovoExpress™, ACEA Bioscience. Inc., USA). In brief, 4T1 cells were seeded in a 6-well plate with a density of 2 × 10^5^ cells per well and incubated in DMEM medium for 24 h. Thereafter, the culture medium was replaced by different formulations including fresh DMEM medium, Alg@CaP, GO, GO-Alg@CaP, Cur, Alg@CaP/C, Obatoclax, Alg@CaP/O, GO-Alg@CaP/C, and GO-Alg@CaP/CO. After co-incubated for another 48 h, cells would be collected and resuspending in PBS after washed thrice. Eventually, samples were stained with Annexin-V-FITC and PI and measured by the flow cytometry.

### In vitro Ca^2+^ detection by CLSM

Fluo-3AM Ca^2+^ fluorescent probe and Rhod-2AM probe were both used to measure Ca^2+^ in cells and mitochondria, respectively. After treated with different formulations (PBS, Alg@CaP, GO, GO-Alg@CaP, Cur, Alg@CaP/C, Obatoclax, Alg@CaP/O, GO-Alg@CaP/C, and GO-Alg@CaP/CO) for 6 h, cells were loaded with probes and then conducted by confocal laser scanning microscope. To further confirm the co-localization of mitochondrial Ca^2+^ and mitochondria, mitochondrial green fluorescent probes were used as well.

### Mitochondrial membrane potential

4T1 cells were seeded on the glass-bottom dish and incubated with different treatments for 24 h. The cells were then stained with JC-1 and washed with PBS for thrice. Confocal laser scanning microscope was used to visualize mitochondrial membrane potential.

### ATP content

In order to detect the intracellular ATP content, different treatments (PBS, Alg@CaP, GO, GO-Alg@CaP, Cur, Alg@CaP/C, Obatoclax, Alg@CaP/O, GO-Alg@CaP/C, and GO-Alg@CaP/CO) were set. 4T1 cells were seeded in a 6-well plate with a density of 2 × 10^5^ cells per well and incubated in DMEM medium for 24 h. Thereafter, the culture medium was replaced by different formulations in 6-well plates for 48 h. A multifunctional microplate reader was established to measure cell lysates by ATP Detection Kit.

### Western blotting assay and bio-TEM of autophagosome

Autophagy related proteins (LC3, P62) were analyzed by Western blot. 4T1 cells were seeded in a 6-well plate at a density of 1 × 10^5^ cells per well and then treated as described above. Afterwards BCA protein kit was used to quantify the harvested cells lysates. According to the standard protocol, the proteins were separated from SDS-PAGE gels and transferred onto a PVDF membrane. To probe protein level, the membrane was blocked and then incubated with primary and secondary antibodies. Different protein bands were visualized by chemo-luminescence imaging system (Bio-Rad, ChemiDoc XR + UV illuminator, USA). For autophagy inhibition analysis, 4T1 cells were incubated with similar treatment above. Cells were washed after incubated for another 48 h and fixed in 2.5% glutaraldehyde in PBS for 4 h at 4 °C. After that, 1% osmium tetroxide in 0.1 M cacodylate buffer was used to fix the cells for 1 h at room temperature. The cells were then embedded in epoxy resin after dehydrated with a graded series of ethanol. 70 nm sections were cut from the epoxy resin and subsequently conducted with 4% uranyl acetate and 2.5% glutaraldehyde staining. Finally, the sections were analyzed through TEM (Additional file 2).

### In vivo biodistribution of GO-Alg@CaP/CO

All animal procedures were performed in compliance with the ethical rules in Beijing. Female BALB/c mice (6–8 weeks-old) were purchased from Vital River Laboratory Animal Center (Beijing, China). The 4T1 xenograft tumor models were established by subcutaneous injecting into the right back of the mice at the density of 1.0 × 10^6^ per mouse. The following imaging and anti-tumor experiments were administrated when the tumor volumes reached approximately 100 mm^3^. The tumor-bearing mice were subcutaneous injected with at the 1 μg/mL concentration of IR780. At different time-points post injection, the images were then captured with multi-spectral fluorescence (Cri-M2, CRI USA) imaging system. After that, mice were sacrificed at 48 h and various tissues (heart, liver, spleen, lung, kidney, and tumor) were then harvested and washed twice with saline before ex vivo fluorescence imaging.

### In vivo antitumor assay

4T1 tumour-bearing mice were randomly divided into 10 groups and post injected with different formulations (GO dose: 2 mg/kg, Cur dose: 10 mg/kg, Obatoclax dose: 0.25 mg/kg). Tumor weight and volumes were recorded per 2 days. For systemically evaluating the biosafety of various operations, serum was collected for blood biochemical analysis (Charles River Laboratories, Beijing, China) after treatment. The main tissues (heart, liver, spleen, lung, kidney, and tumor) were dissected with H&E staining and tumor tissues also stained with TUNEL assays.

## Supplementary Information

**Additional file 1: Table S1.** The average sizes, zeta potentials and polydispersity index (PDI) of GO-Alg@CaP with different ratios of GO-Alg/CaP. **Table S2.** The average sizes, zeta potentials and polydispersity index (PDI) of different preparations. **Figure S1.** The FT-IR spectra of CaP, Alg, Alg@CaP, GO, GO-Alg and GO-Alg@CaP/CO. **Figure S2.** The standard curves of glucose according to the DNS assay. **Figure S3.** Particle size A and zeta potentials B changes of GO-Alg@CaP/CO measured by dynamic light scattering (DLS) at pH 7.4 for 7 days. **Figure S4.** Release profiles of A Obatoclax and B Curcumin from GO-Alg@CaP/CO in pH 7.4 and pH 5.2. **Figure S5.** CLSM images of 4T1 cells incubated with Cur for 0.5 h, 2 h and 6 h. The lysosome and nucleus were stained with Lyso-Tracker Red and Hoechst 33342, respectively. The scale bar was 50 μm. **Figure S6.** CLSM images of 4T1 cells incubated with GO-Alg@CaP/C for 0.5 h, 2 h and 6 h. The lysosome and nucleus were stained with Lyso-Tracker Red and Hoechst 33342, respectively. The scale bar was 50 μm. **Figure S7.** Mitochondrial membrane potentials and distributions of 4T1 cells treated with different preparations for 24 h. Scale bars were 50 μm. **Figure S8.** Hemolysis assay with different concentrations of Alg@CaP (negative control: phosphate buffer solution; positive control: water). **Figure S9.** Individual tumor growth curves of the mice in different groups for 15-day treatments. **Figure S10.** H&E-stained tumor slices collected from 4T1 tumor-bearing mice after treatments for 15 days. **Figure S11.** Photographs of tumors ex vivo in different treatment groups within 15 days. **Figure S12.** The ATP content in tumors detected by ATPlite Assay Kit after 15 days GO-Alg@CaP/CO treatment. Data are mean ± SD, n = 3; *P < 0.05, **P < 0.01 and ***P < 0.001. **Figure S13.** P62, LC3-I, and LC3-II expression in tumor after 14 days treatments of GO-Alg@CaP and GO-Alg@CaP/CO. **Figure S14.** H&E staining images of major organs collected from mice under various treatments after 15 days. The scale bar was 200 nm. **Figure S15.** Blood analysis of mice at 24 h and 7 days after GO-Alg@CaP/CO injection. **Figure S16.** Relative quantification ratio of CD4^+^ and CD8^+^ gating on CD3^+^ cells. **Figure S17.** In vivo fluorescence images of 4T1 tumor-bearing mice that were intravenously injected with GO-Alg@CaP/I at different time points for three repeated experiments.

**Additional file 2.** Raw western blot results for three repeats.

## Data Availability

Without restrictions.
