# Supplementary material for: Boosting nutrient starvation-dominated cancer therapy through curcumin-augmented mitochondrial Ca2+ overload and obatoclax-mediated autophagy inhibition as supported by a novel nano-modulator GO-Alg@CaP/CO
Source: J Nanobiotechnology. 2022 May 12;20:225. doi: 10.1186/s12951-022-01439-0 (PMC9097046; doi:10.1186/s12951-022-01439-0)
Supplement: Supplementary file 1 — Additional file 1: Table S1. The average sizes, zeta potentials and polydispersity index (PDI) of GO-Alg@CaP with different ratios of GO-Alg/CaP. Table S2. The average sizes, zeta potentials and polydispersity index (PDI) of different preparations. Figure S1. The FT-IR spectra of CaP, Alg, Alg@CaP, GO, GO-Alg and GO-Alg@CaP/CO. Figure S2. The standard curves of glucose according to the DNS assay. Figure S3. Particle size A and zeta potentials B changes of GO-Alg@CaP/CO measured by dynamic light scattering (DLS) at pH 7.4 for 7 days. Figure S4. Release profiles of A Obatoclax and B Curcumin from GO-Alg@CaP/CO in pH 7.4 and pH 5.2. Figure S5. CLSM images of 4T1 cells incubated with Cur for 0.5 h, 2 h and 6 h. The lysosome and nucleus were stained with Lyso-Tracker Red and Hoechst 33342, respectively. The scale bar was 50 μm. Figure S6. CLSM images of 4T1 cells incubated with GO-Alg@CaP/C for 0.5 h, 2 h and 6 h. The lysosome and nucleus were stained with Lyso-Tracker Red and Hoechst 33342, respectively. The scale bar was 50 μm. Figure S7. Mitochondrial membrane potentials and distributions of 4T1 cells treated with different preparations for 24 h. Scale bars were 50 μm. Figure S8. Hemolysis assay with different concentrations of Alg@CaP (negative control: phosphate buffer solution; positive control: water). Figure S9. Individual tumor growth curves of the mice in different groups for 15-day treatments. Figure S10. H&E-stained tumor slices collected from 4T1 tumor-bearing mice after treatments for 15 days. Figure S11. Photographs of tumors ex vivo in different treatment groups within 15 days. Figure S12. The ATP content in tumors detected by ATPlite Assay Kit after 15 days GO-Alg@CaP/CO treatment. Data are mean ± SD, n = 3; *P < 0.05, **P < 0.01 and ***P < 0.001. Figure S13. P62, LC3-I, and LC3-II expression in tumor after 14 days treatments of GO-Alg@CaP and GO-Alg@CaP/CO. Figure S14. H&E staining images of major organs collected from mice under various treatments afte [file 12951_2022_1439_MOESM1_ESM.docx]

**Additional file 1**

**
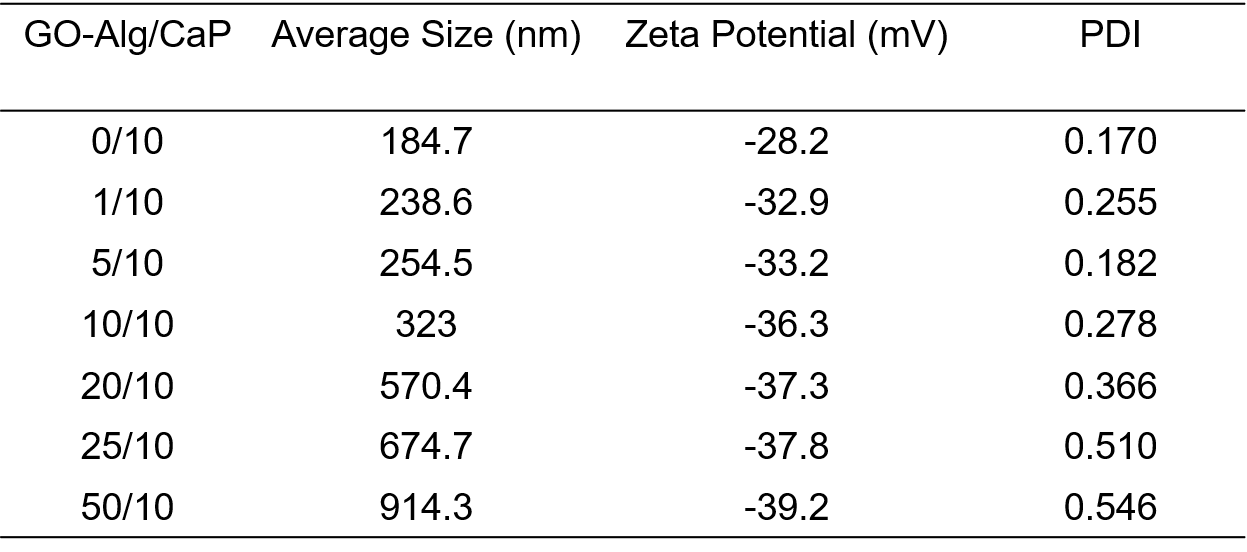
**

**Table S1**. The average sizes, zeta potentials and polydispersity index (PDI) of GO-Alg@CaP with different ratios of GO-Alg/CaP.

**
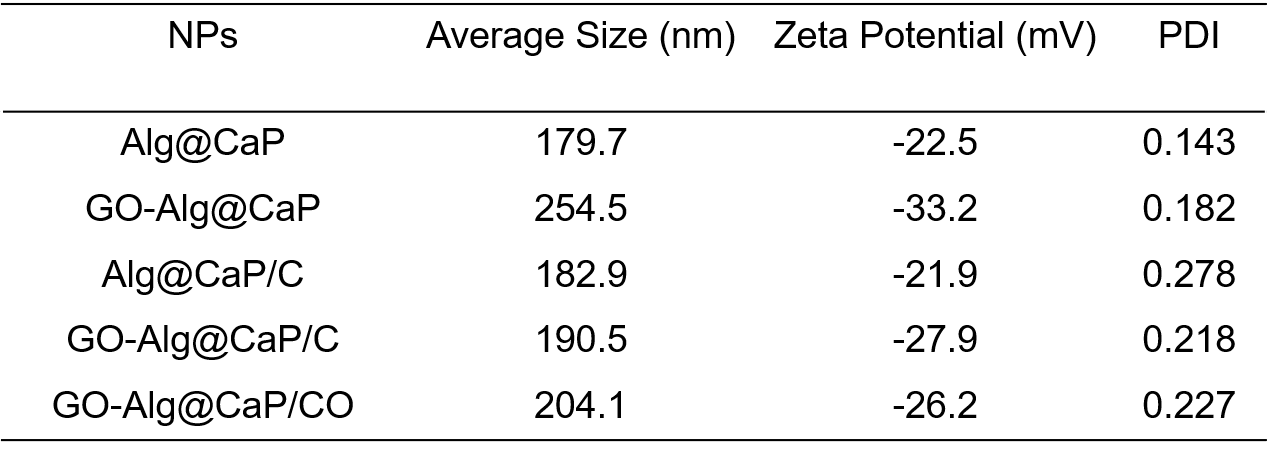
**

**Table S2**. The average sizes, zeta potentials and polydispersity index (PDI) of different preparations.

**
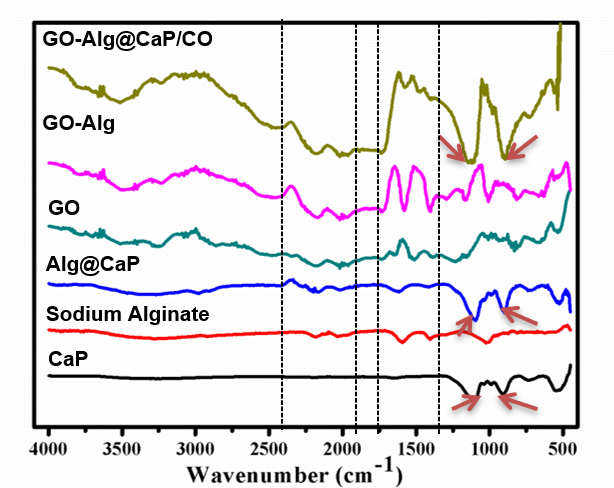
**

**Fig S1**. The FT-IR spectra of CaP, Alg, Alg@CaP, GO, GO-Alg and GO-Alg@CaP/CO.


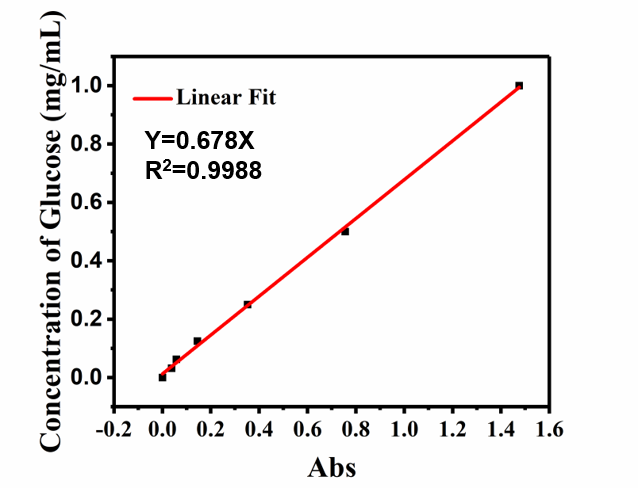


**Fig S2**. The standard curves of glucose according to the DNS assay.

**Fig S3**. Particle size **A** and zeta potentials **B** changes of GO-Alg@CaP/CO measured by dynamic light scattering (DLS) at pH 7.4 for 7 days.

**Fig S4**. Release profiles of **A** Obatoclax and **B** Curcumin from GO-Alg@CaP/CO in pH 7.4 and pH 5.2.

**
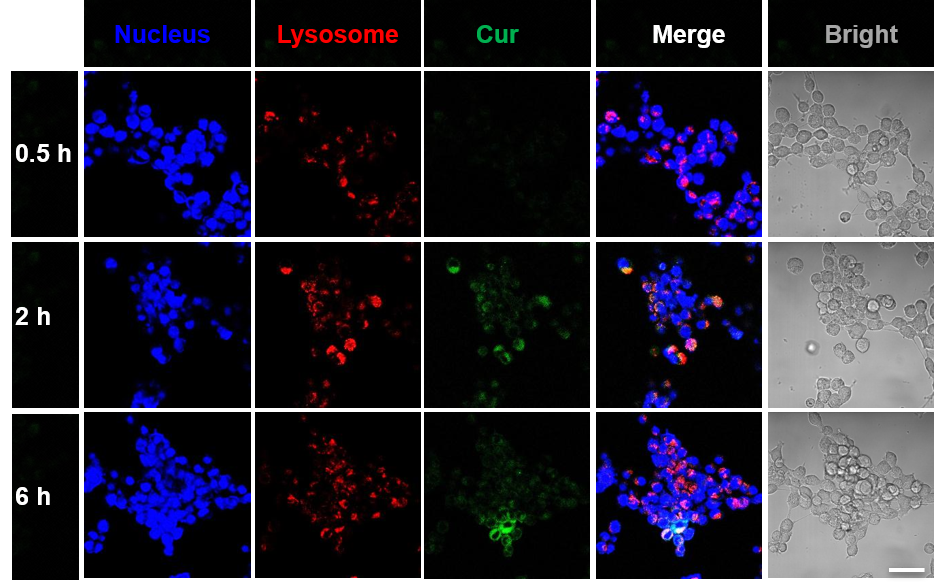
**

**Fig S5**. CLSM images of 4T1 cells incubated with Cur for 0.5h, 2h and 6 h. The lysosome and nucleus were stained with Lyso-Tracker Red and Hoechst 33342, respectively. The scale bar was 50 μm.

**
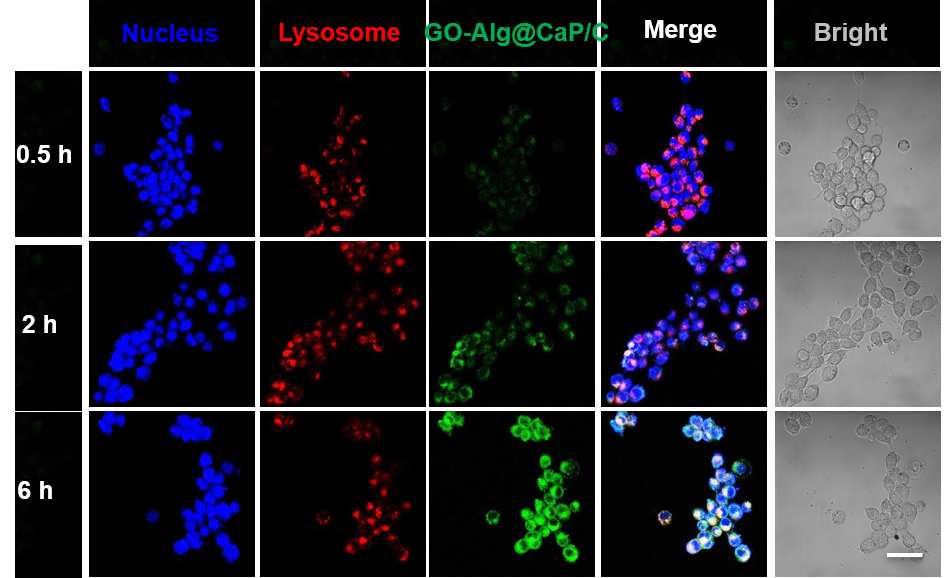
Fig S6**. CLSM images of 4T1 cells incubated with GO-Alg@CaP/C for 0.5h, 2h and 6 h. The lysosome and nucleus were stained with Lyso-Tracker Red and Hoechst 33342, respectively. The scale bar was 50 μm.

**Fig S7** Mitochondrial membrane potentials and distributions of 4T1 cells treated with different preparations for 24 h. Scale bars were 50 μm.


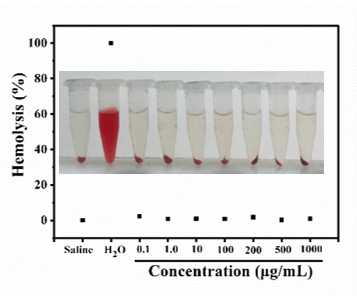


**Fig S8**. Hemolysis assay with different concentrations of Alg@CaP (negative control: phosphate buffer solution; positive control: water).

**
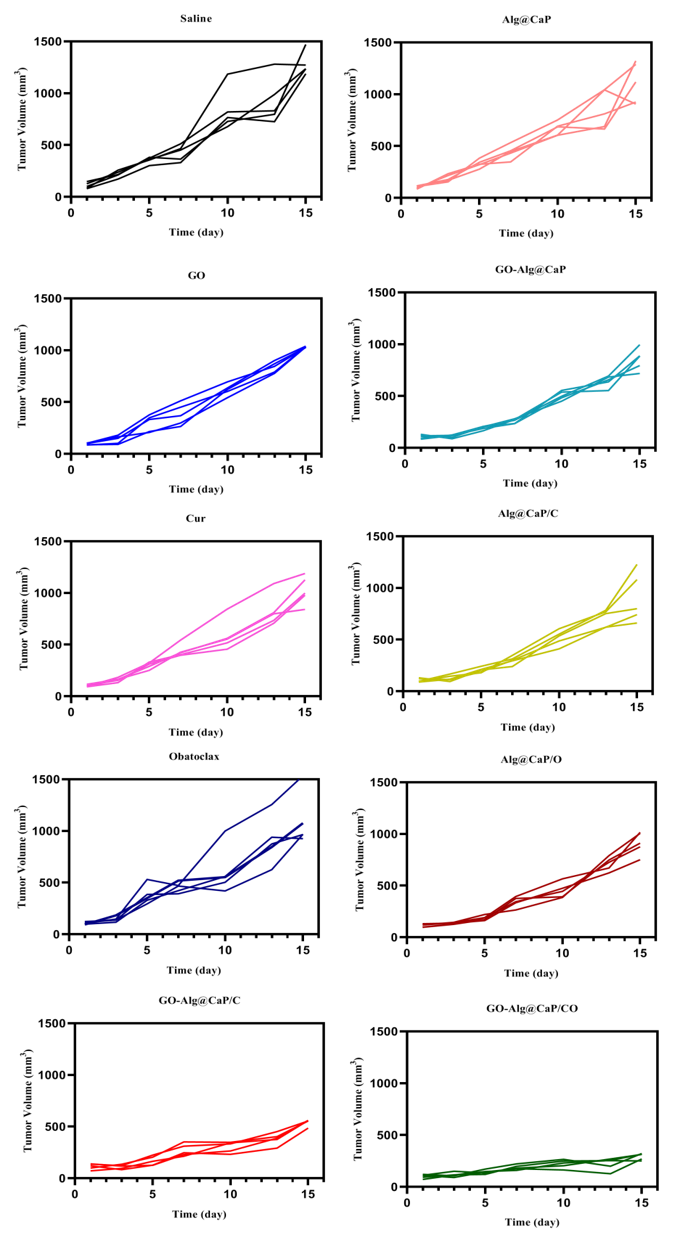
**

**Fig S9**. Individual tumor growth curves of the mice in different groups for 15-day treatments.


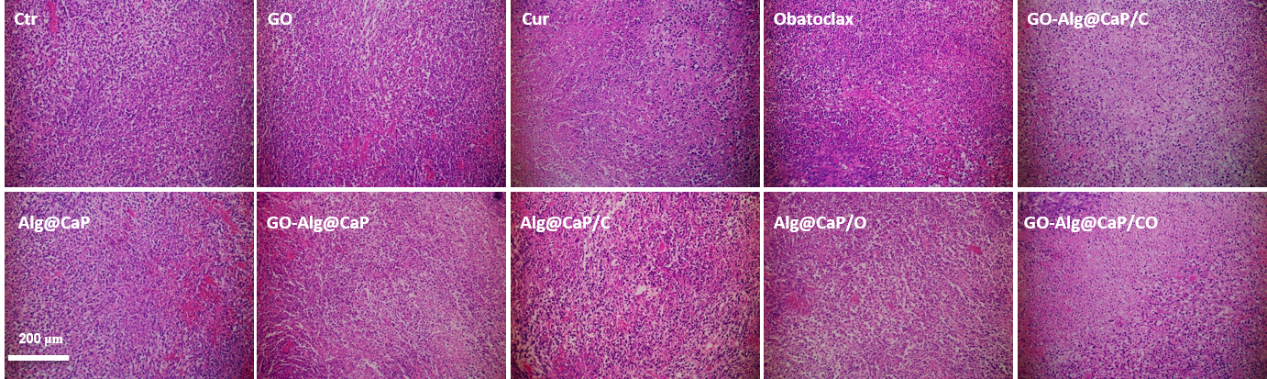


**Fig S10**. H&E-stained tumor slices collected from 4T1 tumor-bearing mice after treatments for 15 days.

**
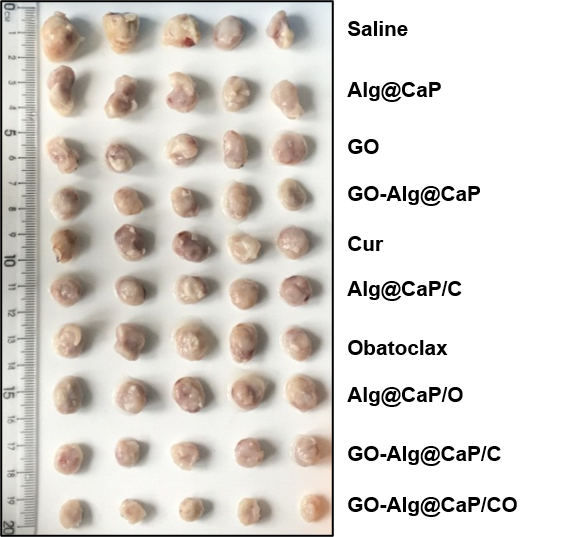
**

**Fig S11**. Photographs of tumors ex vivo in different treatment groups within 15 days.

**Fig S12**. The ATP content in tumors detected by ATPlite Assay Kit after 15 days GO-Alg@CaP/CO treatment. Data are mean ±SD, n=3; *P<0.05, **P<0.01 and ***P<0.001.

**Fig S13**. P62, LC3-I, and LC3-II expression in tumor after 14 days treatments of GO-Alg@CaP and GO-Alg@CaP/CO.

**Fig S14.** **H&E staining images of major organs collected from mice under various treatments after 15 days. The scale bar was 200 nm.**

**Fig S15. Blood analysis of mice at 24 h and 7 days after GO-Alg@CaP/CO injection.**

**Fig S16. Relative quantification ratio of CD4^+^ and CD8^+^ gating on CD3^+^ cells**

**Fig S17**. In vivo fluorescence images of 4T1 tumor-bearing mice that were intravenously injected with GO-Alg@CaP/I at different time points for three repeated experiments.
