# Supplementary material for: Boosting nutrient starvation-dominated cancer therapy through curcumin-augmented mitochondrial Ca2+ overload and obatoclax-mediated autophagy inhibition as supported by a novel nano-modulator GO-Alg@CaP/CO
Source: J Nanobiotechnology. 2022 May 12;20:225. doi: 10.1186/s12951-022-01439-0 (PMC9097046; doi:10.1186/s12951-022-01439-0)

2021-04-29 00hr 40min 30sec ACTIN

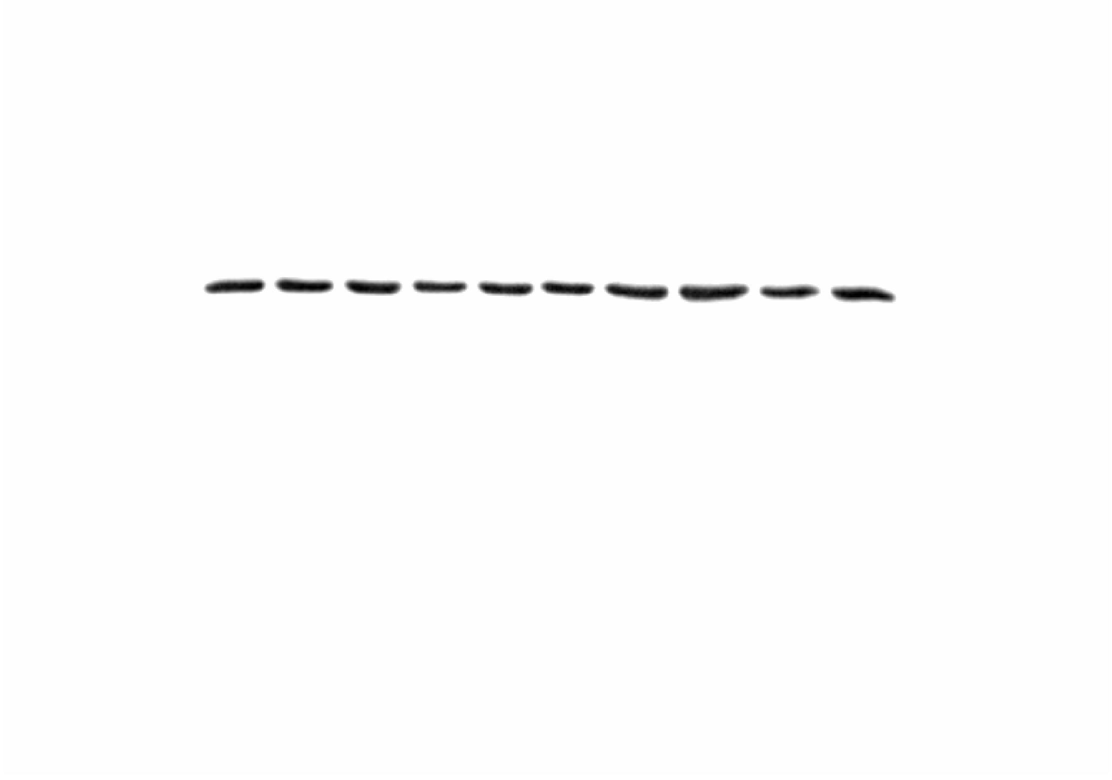

2021-04-29 05hr 40min 38seclc3

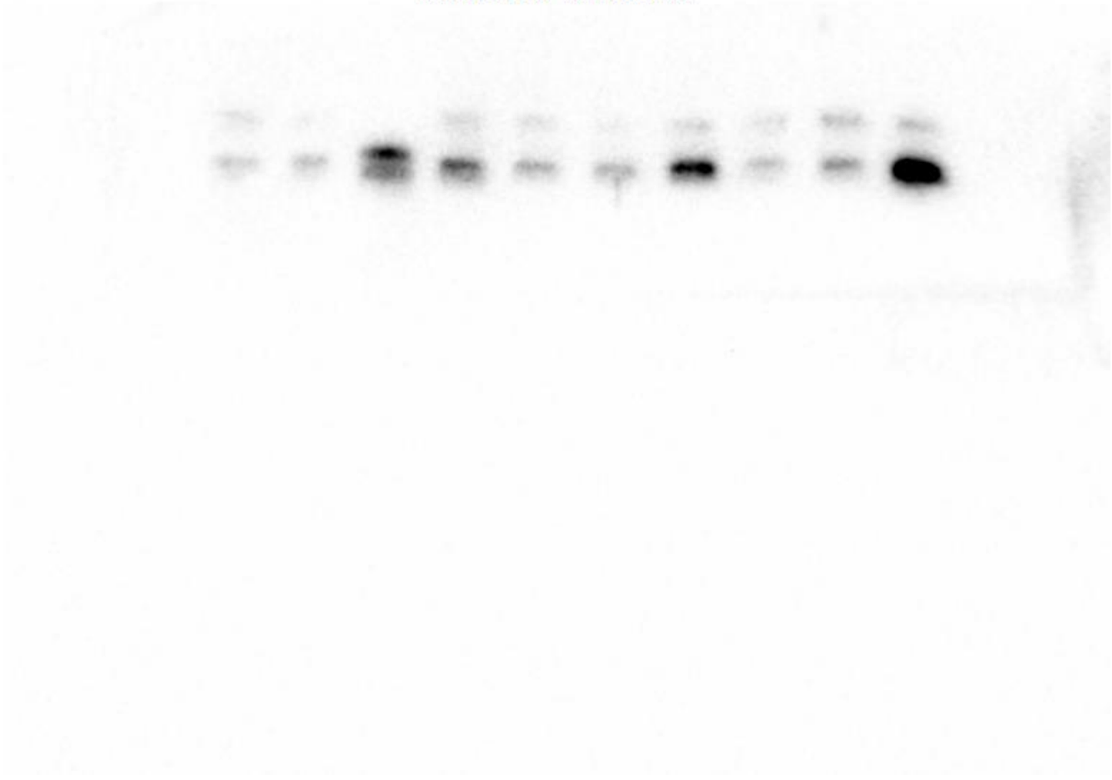

2021-04-29 05hr 43min 08secp62

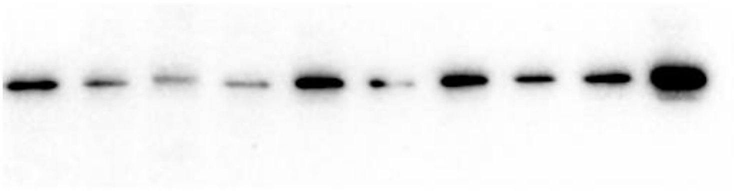

2021-05-17 00hr 03min 57sec actin

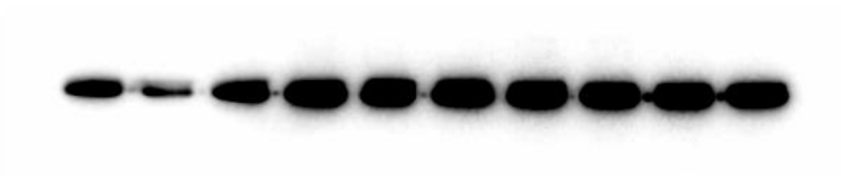

2021-05-17 00hr 05min 52sec lc3

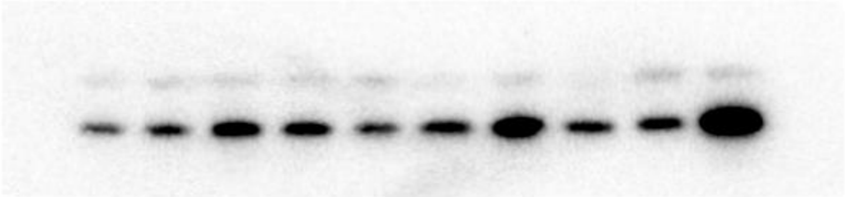

2021-05-17 00hr 10min 18sec p62

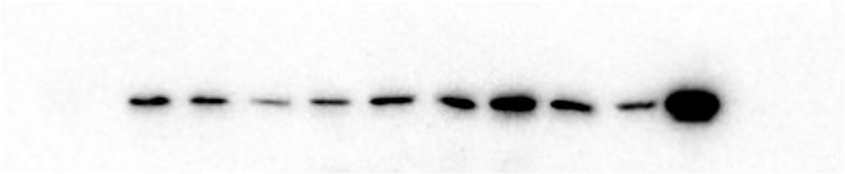

2021-04-02 02hr 11min 18secactin

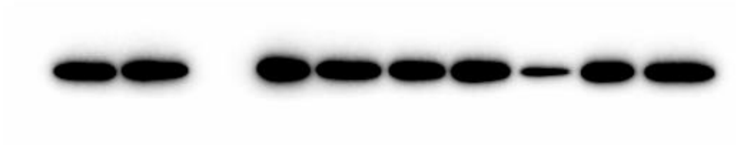

2021-04-02 02hr 06min 21sec lc3

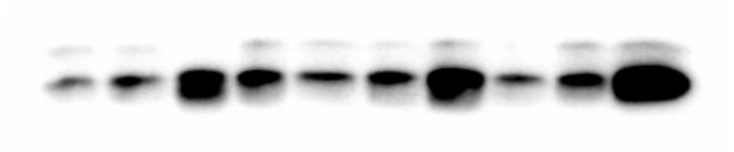

2021-04-02 02hr 09min 43sec p62

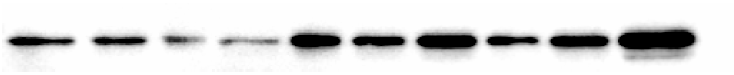

Supplement: Supplementary file 2 — Additional file 2. Raw western blot results for three repeats. [file 12951_2022_1439_MOESM2_ESM.pdf]
